# Antimicrobial resistance (AMR) and molecular characterization of *Neisseria gonorrhoeae* in Ghana, 2012-2015

**DOI:** 10.1371/journal.pone.0223598

**Published:** 2019-10-10

**Authors:** Naiki Attram, Bright Agbodzi, Helena Dela, Eric Behene, Edward O. Nyarko, Nicholas N. A. Kyei, John A. Larbi, Bernard W. L. Lawson, Kennedy K. Addo, Mercy J. Newman, Christopher A. Duplessis, Nehkonti Adams, Magnus Unemo, Andrew G. Letizia

**Affiliations:** 1 US Naval Medical Research Unit Number Three, Ghana Laboratory, Legon, Ghana; 2 Noguchi Memorial Institute for Medical Research, University of Ghana, Legon, Ghana; 3 Department of Public Health, Military Hospital, Accra, Ghana; 4 Department of Theoretical and Applied Biology, Kwame Nkrumah University of Science and Technology, Kumasi, Ghana; 5 Department of Medical Microbiology, School of Biomedical and Allied Health Science, College of Health Sciences, University of Ghana, Accra, Ghana; 6 WHO Collaborating Centre for Gonorrhoea and other STIs, National Reference Laboratory for STIs, Department of Laboratory Medicine, Faculty of Medicine and Health, Örebro University, Örebro, Sweden; Emory University School of Medicine, UNITED STATES

## Abstract

*Neisseria gonorrhoeae* antimicrobial resistance (AMR) surveillance is essential for tracking the emergence and spread of AMR strains in local, national and international populations. This is crucial for developing or refining treatment guidelines. *N*. *gonorrhoeae* multiantigen sequence typing (NG-MAST) is beneficial for describing the molecular epidemiology of gonococci at national and international levels. Elucidation of AMR determinants to β-lactam drugs, is a means of monitoring the development of resistance. In Ghana, little is known about the current gonococcal AMR prevalence and no characterization of gonococcal isolates has been previously performed. In this study, gonococcal isolates (n = 44) collected from five health facilities in Ghana from 2012 to 2015, were examined using AMR testing, NG-MAST and sequencing of *penA*. High rates of resistance were identified to tetracycline (100%), benzylpenicillin (90.9%), and ciprofloxacin (81.8%). One isolate had a high cefixime MIC (0.75 μg/ml). Twenty-eight NG-MAST sequence types (STs) were identified, seventeen of which were novel. The isolate with the high cefixime MIC contained a mosaic *penA*-34 allele and belonged to NG-MAST ST1407, an internationally spreading multidrug-resistant clone that has accounted for most cefixime resistance in many countries. In conclusion, AMR testing, NG-MAST, and sequencing of the AMR determinant *penA*, revealed high rates of resistance to tetracycline, benzylpenicillin, and ciprofloxacin; as well as a highly diverse population of *N*. *gonorrhoeae* in Ghana. It is imperative to continue with enhanced AMR surveillance and to understand the molecular epidemiology of gonococcal strains circulating in Ghana and other African countries.

## Introduction

Antimicrobial resistance (AMR) in *Neisseria gonorrhoeae* is an escalating global public health problem [1–5]. This has been exacerbated in many African countries, including Ghana, by a high burden of gonorrhoea but limited etiological case detection. The World Health Organization (WHO) recommends dual antimicrobial therapy over single therapy to treat gonorrhoea in settings where appropriate up-to-date local AMR data is unavailable [4]. AMR data for *N*. *gonorrhoeae* in Ghana is rare, thus, syndromic management of gonorrhoea is the norm [6, 7]. The published Standard Treatment Guidelines for 2018 by the Ministry of Health in Ghana recommend a single dose of one of the following: 250 mg intramuscular injection of ceftriaxone or 400 mg oral cefixime or 500 mg oral ciprofloxacin, combined with one of the following treatments for *Chlamydia trachomatis* infection: 100 mg oral doxycycline, twice daily for 7 days or 500 mg oral tetracycline, four times daily for 7 days or 500 mg oral erythromycin, four times daily for 7 days or 1 g of oral azithromycin [8]. In recent years, we have established gonococcal AMR surveillance in selected sentinel clinics in Ghana [9].

*N*. *gonorrhoeae* multiantigen sequence typing (NG-MAST) has been used in the molecular studies of *N*. *gonorrhoeae* in many countries [10–12]. The method is particularly suited for answering short term epidemiological questions by identifying circulating strains and providing information on their evolution and dissemination [13].

Decreased susceptibility and resistance to ESCs is mainly due to mosaic *penA* alleles and other specific mutations in the *penA* gene that encodes the penicillin binding protein 2 (PBP2). These genetic polymorphisms have been shown to cause an increase in the MICs of cefixime and ceftriaxone as well as treatment failure [14–17].

The aims of the present study were to examine the AMR of *N*. *gonorrhoeae* isolates collected from three coastal cities in Ghana (Accra, Sekondi and Takoradi) during 2012 to 2015, describe their NG-MAST sequence types (STs), and sequence their ESC resistance determinant *penA*. This data would also be a first step towards including Ghana in the WHO GASP. Preliminary data describing the AMR of seven and six of the 44 isolates characterized in the present study were included in a published paper and short report, respectively [9, 18].

## Materials and methods

### Biological sample and data collection

Patients presenting to five health facilities were selectively identified based on their clinical symptoms. These included: vaginal discharge, dysuria, intermenstrual bleeding, or abdominal pain in females or urethral discharge/ dysuria in males. Study volunteers were enrolled in the study after written informed consent was obtained as previously described [18]. An endocervical or urethral swab from each participant was collected and immediately inoculated on modified Thayer Martin media (MTM) agar. Promptly after inoculation, the agar plate was placed in a BD gas pack EZ-CO2 pouch system (Becton Dickinson, New Jersey, USA), which was then put in a cool box at ambient temperature (between 25°C and 30°C) for immediate transport to the laboratory. Demographic (age, gender, and marital status) and risk behavior data (sexual practices, alcohol use and condom use) were collected via a structured questionnaire.

### *N*. *gonorrhoeae* culture and species identification

In the laboratory, each inoculated plate was incubated at 35–37°C for 18–72 hours in a humid, 5% CO_2_-enriched atmosphere. Plates were checked for growth every day; any isolate showing Gram-negative diplococci by microscopy was re-cultured under the same conditions on non-selective GC base chocolate agar with 1% Isovitalex (Becton Dickinson, USA). Growth was subsequently tested for rapid catalase and oxidase reactivity as well as with the API-NH (Biomerieux, Marcy l’Etoile, France) test for species identification of *N*. *gonorrhoeae*. All isolates were preserved in tryptic soy broth (TSB) supplemented with 20% glycerol at -70°C.

### Antimicrobial susceptibility testing

Disk diffusion methods were used to assess susceptibility to ceftriaxone, cefixime, ciprofloxacin, tetracycline, azithromycin, benzylpenicillin and spectinomycin of all the isolates. Resistance was then confirmed with the Etest (Biomerieux, Marcy l’Etoile, France), except for an arbitrary collection of isolates which most likely lost their viability due to power fluctuations. The media used for these assays were GC agar supplemented with 1% isovitalex and 1% heme. Inhibition zone diameters (mm) and minimum inhibitory concentration (MIC; μg/ml) values were used to determine susceptibility (S), intermediate susceptibility (I) or resistance (R) using breakpoints recommended by the CLSI [19]. Breakpoints for azithromycin are not stated by the CLSI thus, ˂30 mm was used as the disc diffusion breakpoint and ˃1 μg/ml was the MIC breakpoint value for resistance, as described earlier [20]. The API-NH kit was used to determine ß-lactamase activity of each isolate. Quality control of bacteriological procedures was performed using the *N*. *gonorrhoeae* reference strain ATCC 49226.

### Genetic characterization

DNA extracts from *N*. *gonorrhoeae* isolates were obtained with the QIAmp DNA extraction kit (Qiagen, USA), according to manufacturer’s instructions. NG-MAST and sequencing of *penA* were performed as previously described [10, 21, 22]. Purified PCR products were sequenced with an ABI genetic analyzer (Applied Biosystems, Perkin Elmer USA).

#### Sequence alignments and phylogenetic analysis

At the NG-MAST website, (http://www.ng-mast.net/), the NG-MAST-trimmed *porB* (490 bp) and *tbpB* (390 bp) gene sequences were assigned allele numbers and STs based on the allelic profile of each isolate. The *porB* and *tbpB* allele sequences of each isolate were then concatenated with the help of the FASconCAT-G software package [23]. After aligning the concatenated sequences, evolutionary relationships between the isolates were investigated by neighbor joining phylogenetic analysis on the MEGA6 platform [24].

The *penA* allele fragments were assembled with the CodonCode Aligner version 7.1.2 software (CodonCode Corporation, USA), alongside a reference wild-type *penA* gene sequence with GenBank accession no. M32091. The *penA* sequences were assigned allele numbers based on the *N*. *gonorrhoeae* Sequence Typing Antimicrobial Resistance (NG-STAR) nomenclature [25]. Sequence analysis was performed with the MEGA version 6 software package [24].

### Statistical analysis

Percentage calculations were performed to determine resistance rates and rates of occurrence of the different STs. Fishers’ exact testing was used to determine association between STs and antimicrobial resistance/susceptibility, as well as association between STs and select risk factors or demographic information.

**Ethical considerations**: Written informed consent was obtained from each study participant. This study was approved by the Institutional Review Boards of the Naval Medical Research Center, Maryland (approval number: NAMRU3.2012.0007), the 37 Military Hospital, Ghana (approval number: IRB IPN 085/2017), the Noguchi Memorial Institute for Medical Research, Ghana (approval number: NMIMR IRB CPN 054/11-12) and the Ghana Health Service Ethics Review Committee, Ghana (approval number: GHS ERC 03/11/2012).

## Results

### Demographic data

A total 44 gonococcal isolates obtained from 43 males and one female were analyzed. The total number of males and females that were enrolled was 411 and 579 respectively. Most (54.5%) isolates were cultured from patients aged 25–31 years, followed by those aged 16–24 years (29.5%), those aged 32–38 years (9.1%) and those aged ≥46 years (4.4%) (Table 1).

**Table 1:** Antimicrobial resistance determined by the Etest in *Neisseria gonorrhoeae* isolates from 44 patients.

	TET N (%)	PEN N (%)	CIP N (%)	CFM^d^ N (%)	AZM ^c^ N (%)	CRO ^e^ N (%)	SPT N (%)	β-lactamase N (%)
**Number of resistant isolates**	44 (100)	40 (90.9)	36 (81.8)	1 (2.2)	0 (0)	0 (0)	0 (0.0)	36 (81.8)
**Gender (n)**								
Male (N = 43)	43 (100)	39 (90.7)	35 (79.5)	1 (2.3)	0 (0.0)	0 (0.0)	0 (0.0)	35 (81.4)
Female (N = 1)	1 (100.0)	1 (100.0)	1 (100.0)	0 (0.0)	0 (0.0)	0 (0.0)	0 (0.0)	1 (100)
**Age, years (N)**^b^								
18–24 (N = 13)	13 (100)	13 (100.0)	11 (84.6)	0 (0.0)	0 (0.0)	0 (0.0)	0 (0.0)	11 (84.6)
25–31 (N = 24)	24 (100.0)	21 (87.5)	20 (83.3)	1 (4.1)	0 (0.0)	0 (0.0)	0 (0.0)	19 (79.2)
32–38 (N = 4)	4 (100.0)	4 (100.0)	2 (50.0)	0 (0.0)	0 (0.0)	0 (0.0)	0 (0.0)	4 (100)
46 and above (N = 2)	2 (100.0)	1 (50.0)	2 (100.0)	0 (0.0)	0 (0.0)	0 (0.0)	0 (0.0)	1 (50.0)

^a^Antimicrobial resistance was determined with the disk diffusion method as described by the CLSI (CLSI, 2015). Resistant and intermediate susceptible isolates were further confirmed by the Etest, where possible. Data described in this table are all Etest results.

^b^ One response for age was missing.

^c^ It was not possible to use the Etest to examine five isolates showing resistance to azithromycin by the disc diffusion method

^d^ It was not possible to use the Etest to examine one isolate with reduced susceptibility to cefixime by the disc diffusion method.

^e^ It was not possible to use the Etest to examine six isolates that showed reduced susceptibility to ceftriaxone by the disc diffusion method

TET = tetracycline, PEN = penicillin, CIP = ciprofloxacin, CFM = cefixime, AZM = azithromycin, CRO = ceftriaxone, SPT = spectinomycin

### Antimicrobial resistance data

Overall, 100%, 90.9% and 88.6% of the isolates showed *in vitro* resistance to tetracycline, benzylpenicillin and ciprofloxacin, respectively by the disc diffusion method, while by Etest, resistance rates were 100%, 90.9% and 81.8%, respectively (Table 1). The MICs of these resistant isolates ranged from 2 to 256 μg/ml for tetracycline, 3 to ˃32 μg/ml for benzyl penicillin and 3 to ˃32 μg/ml for ciprofloxacin. The majority (79.5%) of isolates had benzylpenicillin MICs of ≥32 μg/ml. The rate of resistance to azithromycin, as determined by the disc diffusion method, was 31.8% (14 isolates). Five of these isolates were not viable for subsequent confirmation by the Etest and the rest (n = 9) were within the following MIC range: 0.094–0.75 μg/ml, and had a median MIC of 0.19 μg/ml. No breakpoints for resistance to cefixime and ceftriaxone are stated by the CLSI, however, inhibition zone sizes of less than 30 mm to cefixime and 35 mm or less to ceftriaxone were detected by the disc diffusion method in 11.4% (n = 5) and 18.2% (n = 8) of the isolates, respectively. Among the five isolates with decreased susceptibility to cefixime by the disc diffusion test, three had MICs of 0.016, 0.094 and 0.125 μg/ml, one had an elevated MIC of cefixime (0.75 μg/ml) and the fifth could not be confirmed because it was not viable. Only two of the eight isolates that demonstrated decreased susceptibility to ceftriaxone by the disc diffusion test were tested and confirmed as susceptible by the Etest (0.023 and 0.004 μg/ml), while the rest could not be confirmed due to loss of viability. Resistance to spectinomycin was not seen.

The isolate with a high MIC of cefixime (MIC = 0.75 μg/ml) was obtained from a 26 year old male in Accra and was resistant to benzylpenicillin (MIC = 4 μg/ml), tetracycline (MIC = 16 μg/ml) and ciprofloxacin (MIC >32 μg/ml). It was however susceptible to azithromycin (MIC = 0.38 μg/ml) and spectinomycin (MIC = 16 μg/ml). The ceftriaxone inhibition zone diameter (35.2 mm) was close to the breakpoint for reduced susceptibility, but this result was one of those that could not be confirmed by Etest.

### Genotypic characterization with NG-MAST

**Fig 1:**
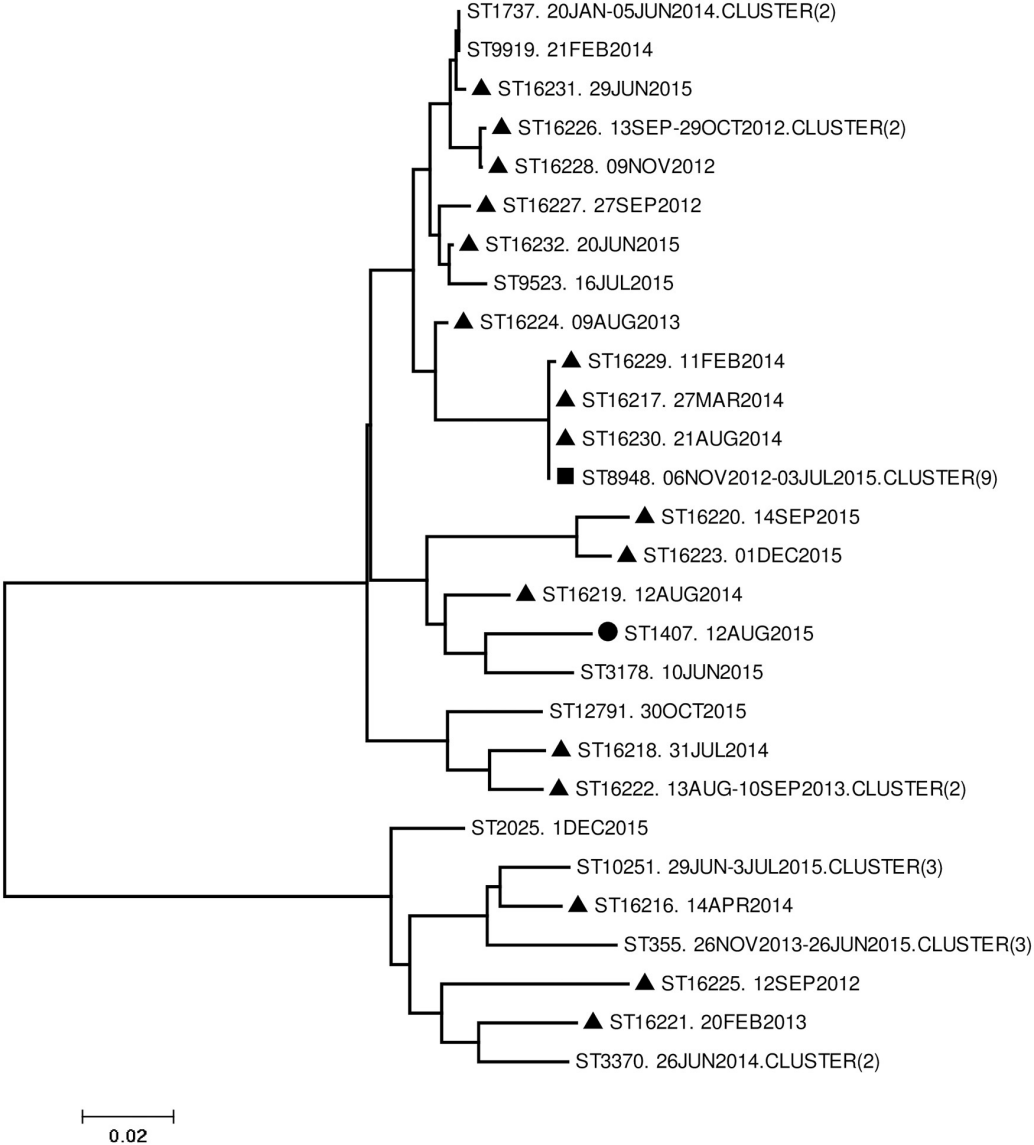
Unrooted neighbor joining phylogram of concatenated *tbpB* and *porB* alleles (880 bp) from *Neisseria gonorrhoeae* isolates. Node labels: Sequence type- Collection date-Single or cluster (number of isolates). **Symbol key**: Square = most abundant sequence type Triangle = novel sequence types. Circle = isolate with high MIC to cefixime.

Molecular analysis was performed on the 44 isolates, NG-MAST STs and corresponding AMR profiles of these isolates are summarized in Table 2. The most frequent ST was ST8948 (9 isolates; 20.5%) followed by ST10251 and ST355 (3 isolates each; 6.8%). Twenty-one (47.7%) isolates had a unique ST (Table 2) whilst novel STs represented 43.2% of isolates. All isolates belonging to the most common sequence types (ST8948, ST10251 and ST355) were resistant to benzylpenicillin, tetracycline and ciprofloxacin. Phylogenetic analysis based on concatenated *porB* and *tbpB* alleles of each different ST, identified one large cluster (13 STs; 23 isolates), and three smaller clusters consisting of 7 (12 isolates), 5 (5 isolates) and 3 (4 isolates) STs (Fig 1). Timelines for collection of the 23 isolates in the large cluster were from 13th September 2012 to 16th July 2015, spanning 34 months. The next cluster was created by 12 isolates collected over 39 months from 12th September 2012 to 1st December 2015. Five isolates collected over 16 months clustered between 12th August 2014 and 1st December, 2015. Finally, the smallest cluster was created over 26 months by 4 isolates from 13th August 2013 to 30th October 2015. The highest average percentage in nucleotide difference between isolates in each cluster was 0.1%. The only isolate with a high MIC of cefixime was assigned to ST1407, and was located on an isolated branch of the phylogenetic tree (Fig 1). No significant association was observed between STs in the above mentioned clusters and age (p = 0.618), gender (p = 1), marital status (p = 0.247), alcohol intake (p = 0.929), condom use (p = 0.480) and sexual activity, defined by the number of sexual partners over the last six months before the infection being investigated (p = 0.453). Additionally, no significant association was detected between any of the above STs and resistance to benzylpenicillin (p = 1.0). Association was not detected between STs in clusters and benzylpenicillin resistant isolates stratified by PPNG and non-PPNG. No association was detected between STs in clusters and tetracycline (p = 0.205). However, association was seen between the STs in clusters and ciprofloxacin resistance (p = 0.018).

**Table 2:** *Neisseria gonorrhoeae* multiantigen sequence types (NG-MAST) of 44 *N*. *gonorrhoeae* isolates obtained in Ghana in 2012–2015, including antimicrobial resistance profile of each sequence type (ST).

Sequence type (ST)	Total N (%)	TET	PEN	CIP	AZM	CFM	CRO	SPT	β-lactamase
**Novel STs**	**19 (43.2)**	**19**	**17**	**14**	**0**	**0**	**0**	**0**	**15**
**16216**	**1**	1	1	0	0	0	0	0	1
**16217**	**1**	1	1	1	0	0	0	0	1
**16218**	**1**	1	1	1	0	0	0	0	1
**16219**	**1**	1	1	0	0	0	0	0	1
**16220**	**1**	1	1	1	0	0	0	0	1
**16221**	**1**	1	1	1	0	0	0	0	0
**16222**	**2**	2	2	0	0	0	0	0	2
**16223**	**1**	1	0	1	0	0	0	0	0
**16224**	**1**	1	1	1	0	0	0	0	1
**16225**	**1**	1	1	0	0	0	0	0	0
**16226**	**2**	2	2	2	0	0	0	0	2
**16227**	**1**	1	1	1	0	0	0	0	1
**16228**	**1**	1	1	1	0	0	0	0	1
**16229**	**1**	1	1	1	0	0	0	0	1
**16230**	**1**	1	0	1	0	0	0	0	0
**16231**	**1**	1	1	1	0	0	0	0	1
**16232**	**1**	1	1	1	0	0	0	0	1
**Known STs**	**25 (56.8)**	**24**	**23**	**22**	**0**	**1**	**0**	**0**	**20**
**8948**	**9 (20.5)**	9	9	9	0	0	0	0	9
**10251**	**3 (6.8)**	3	3	3	0	0	0	0	3
**355**	**3 (6.8)**	3	3	3	0	0	0	0	1
**1737**	**2 (4.5)**	2	1	2	0	0	0	0	1
**3370**	**2 (4.5)**	2	2	1	0	0	0	0	2
**1407**	**1 (2.3)**	0	1	1	0	1	0	0	0
**2025**	**1 (2.3)**	1	1	1	0	0	0	0	1
**3178**	**1 (2.3)**	1	1	0	0	0	0	0	1
**9523**	**1 (2.3)**	1	1	0	0	0	0	0	1
**9919**	**1 (2.3)**	1	0	1	0	0	0	0	0
**12791**	**1 (2.3)**	1	1	1	0	0	0	0	1

#### Molecular analysis of the penA gene

**Fig 2:**
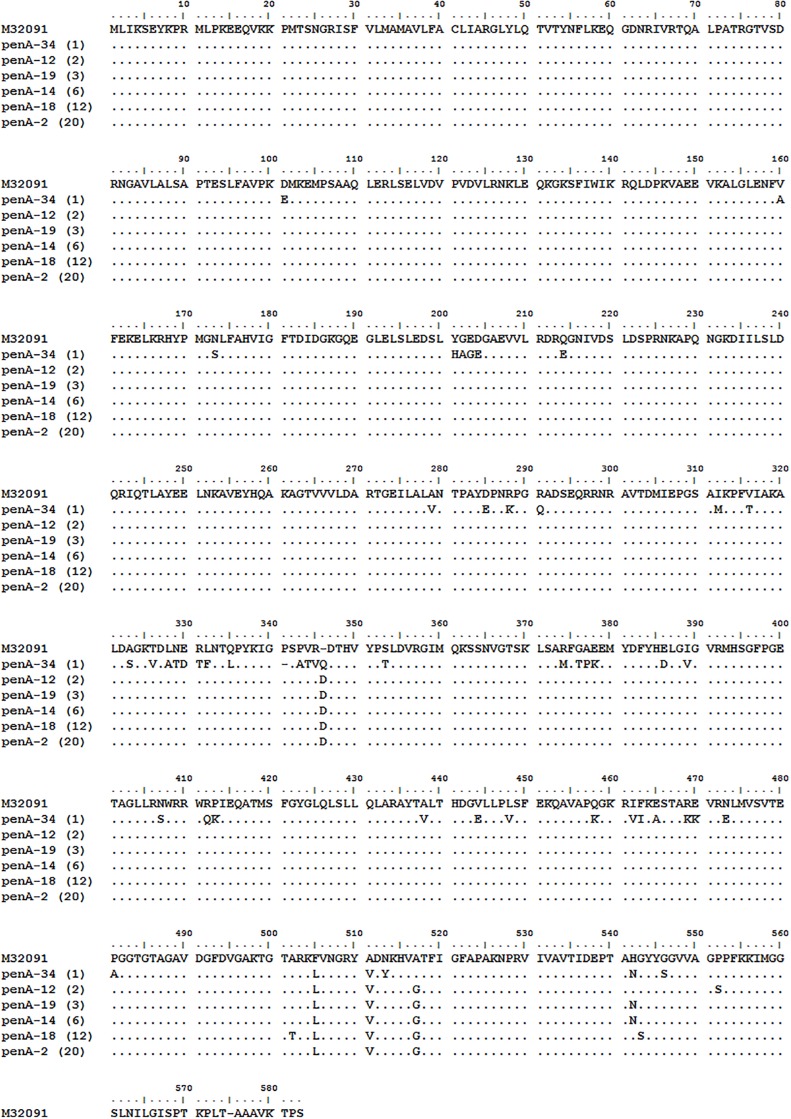
Aligned penicillin binding protein 2 amino acid fragments from 44 *N*. *gonorrhoeae* isolates. Here, the sequences are aligned with a reference sequence (GenBank accession number M32091). Showing in parenthesis is the total number of each pattern that was identified. The dots represent identity with the reference allele, while the dashes represent insertions or deletions.

Six different penA alleles were identified among the 44 isolates Fig 2. The most frequent penA allele was the non-mosaic penA-2 (n = 20), followed by the non-mosaic penA-18 (n = 12), the non-mosaic penA-14 (n = 6), the non-mosaic penA-19 (n = 3), the non-mosaic penA-12 (N = 2) and the mosaic penA-34 (n = 1). The mosaic penA-34 was identified in the NG-MAST ST1407 isolate with a high MIC of cefixime.

## Discussion

We examined 44 gonococcal isolates collected from symptomatic males and females attending five health facilities in Ghana from 2012 to 2015 using AMR testing, NG-MAST and *penA* sequencing. High rates of resistance were identified to tetracycline (100%), benzylpenicillin (90.9%), and ciprofloxacin (81.8%), and one isolate had a very high cefixime MIC (0.75 μg/ml).

A high level of genetic diversity, demonstrated by different NG-MAST sequence types was observed (28 STs in total, among which are 17 novel STs, identified in 44 isolates). Further, twenty-one different STs were identified in single isolates. Molecular epidemiological studies in several other countries over the last five years have showed similar molecular diversity in NG-MAST STs [12, 26–28]. This may be the result of suboptimal diagnostics (several positive cases were missed by culture and only arbitrary gonorrhoea patients and/or isolates were identified), lack of contact tracing (sexual contacts of identified positive cases were not tracked), epidemiological surveillance (sexual transmission chains spreading a single ST were not identified or followed-up), evolution of STs locally in Ghana (STs have not been described in the past because no NG-MAST studies have previously been performed in the country) or STs imported from abroad. The most prevalent ST, ST8948 (nine isolates), was present from the beginning of the surveillance in 2012 and persisted until June 2015. This ST has previously been found in several European countries, but details regarding its first identification are inconclusive. In future gonococcal AMR surveillance in Ghana, whole genome sequencing (WGS) would be a valuable tool to apply. WGS provides substantially improved resolution and accuracy over NG-MAST and can be used to predict AMR as well as identify MDR strains linked to risk groups. WGS has been introduced in international gonococcal AMR surveillance programs [29].

The detection of one isolate with a high MIC of cefixime in Ghana, is worthy of concern. So far in Africa, cefixime resistance associated with cefixime treatment failure has only been described in South Africa [30]. The isolate was found to have a much higher MIC (0.75 μg/ml) than the two South African isolates (0.25 μg/ml), one of which failed to respond to cefixime treatment on two prior occasions. In the case of this Ghanaian isolate, it is not known whether there was treatment failure associated with this strain, or any additional spread of this clone in the country. The NG-MAST ST1407 has been circulating globally and has been responsible for most of the *in vitro* resistance and treatment failures to ESCs (cefixime and ceftriaxone) [31, 32]. This clone, as well as its evolving subclones, has continued to circulate in Europe, USA, Canada and Japan, where it originated [27, 29, 33, 34]. To the best of our knowledge, ST1407 has not been previously reported in any other African country. Unfortunately, the travel history of the patient harboring this strain was not available, and contact tracing was not performed to determine whether it was transferred to anyone else or not.

The six different *penA* alleles identified in this study comprised one mosaic type and five non-mosaic types which have all been identified previously [21, 35, 36]. The non-mosaic alleles all had a D (aspartic acid) insertion between position 346 and 347 of the wild-type *penA* gene. This mutation results in low affinity to penicillin [37, 38]. The mosaic *penA*-34 allele contained polymorphisms that resulted in the following amino acid changes I312M, V316T, N512Y and G545S. These changes have been associated with intermediate penicillin resistance as well as a 10-fold increase in the MIC of ESCs, especially cefixime [15, 36, 37]. Notably, the amino acid alterations in *penA*-12 (P551S) and *penA*-18 (G542S) are also known to contribute to increased MICs of ESCs [39, 40]. Two isolates had the *penA*-12 alteration described above with inhibition zone sizes of 42.2 mm and 30.9 mm to ceftriaxone as well as 38.7 mm and 23.5 mm to cefixime. The second isolates’ inhibition zone diameters to both ceftriaxone and cefixime indicated decreased susceptibility, however its MIC to cefixime was 0.125 μg/ml. The MIC to ceftriaxone could not be determined before the sample lost viability. The disc diffusion inhibition zone diameters to ceftriaxone of the *penA-*18 isolates (n = 12) ranged from 33.3 mm to 47.5 mm. One isolate showed decreased susceptibility by disc diffusion with 33.3 mm, but the MIC was 0.023 μg/ml, i.e. fully susceptible. The rest of the isolates were in the susceptibility range for inhibition zone diameter and were therefore not tested by the Etest. Cefixime inhibition zone diameters of the *penA*-18 isolates ranged from 29.9 mm to 43.6 mm. One isolate had an inhibition zone diameter below the susceptibility breakpoint (29.9 mm), but its MIC could not be determined before it became unviable. The other isolates were not tested by the Etest because their disc diffusion inhibition zone sizes were above the cut off value for susceptibility.

All seven drugs examined in this study are currently in use or have been used in the past to treat gonorrhoea in Ghana. Resistance rates to benzylpenicillin, tetracycline and ciprofloxacin were all above 80%, which is a concern because a few participants reported having used these drugs to treat their gonorrhoea episodes, without a prescription. Self-medication seems to be a common practice, with most people seeking medical attention only when their personal efforts have failed.

The disc diffusion method for AMR testing is used in most Ghanaian clinical settings when available. It is cheap, easy to use and validated in many laboratories worldwide [41]. Compared to Etest and agar dilution methods for MIC determination, disc diffusion methods have been reported to be less reliable and cannot detect small changes in the MIC of antimicrobials [29]. Results of this study support this assertion as interpretations of disc diffusion breakpoint values of some isolates were different from the Etest breakpoint interpretations. For azithromycin susceptibility testing, isolates that were determined to be resistant based on the disc diffusion breakpoint values, were susceptible by the Etest method. The following values were obtained: 29mm, 25mm, 20.8mm, 29.4mm, 27.1mm, 27.8mm, 26.4mm, 28 and 28.1 mm versus 0.75 μg/ml, 0.094 μg/ml, 0.094 μg/ml, 0.38 μg/ml, 0.38 μg/ml, 0.125 μg/ml, 0.125 μg/ml and 0.19 μg/ml. However, replacing the disc diffusion method with the Etest in clinical and laboratory settings in Ghana would be difficult due to its comparatively low price. Nevertheless, future gonococcal AMR surveillance would rely more heavily on Etest or agar dilution methods, which would substantially improve the comparability of data internationally.

Data on AMR trends to *N*. *gonorrhoeae* in Ghana and other African nations are very limited. However, available information indicates that resistance to fluoroquinolones is widespread, as has been detected over the last few years by surveillance efforts in other African countries [42–43]. Antimicrobial resistance in *N*. *gonorrhoeae* needs to be addressed with increased AMR surveillance, optimizations of gonorrhoea treatment guidelines, educational campaigns and stringent stewardship of antimicrobial drug use.

## Limitations of the study

The present study was conducted under material, logistical and technical limitations, leading to the collection of a number of isolates that was too low to make more conclusive determinations. This initial Ghanaian AMR and sequencing data from an ongoing gonococcal surveillance helped identify several areas for improvement which are being addressed in order to improve on isolate yield across board. These areas of improvement include suboptimal sample collection, transportation and storage procedures, and unstable power supply. In order not to lose isolates to temperature fluctuations, attempts would be made to freeze-dry gonococcal isolates. Consistent monitoring and strict adherence to procedures would ensure that collection processes improve. Samples for this study were collected in only the Southern part of Ghana. A nationwide coverage of sentinel sites will help to provide information that is more nationally applicable. Only one isolate was obtained from a female, as compared to isolates from 43 (97.8%) males during the same period. The male dominance may be attributable to challenges posed by endocervical sample collection methods, requiring experience, as well as contamination from bacterial flora in the female genital tract.

Finally, the use of GC agar supplemented with 1% isovitalex and hemoglobin in this study for the disc diffusion and Etest assays resulted in comparatively higher MIC values than were seen in a partner laboratory that made use of the agar dilution assay with GC agar supplemented with isovitalex. This can be seen in a 6-fold MIC difference at both laboratories between the isolate with a high cefixime (0.125 μg/ml versus 0.75 μg/ml). Despite the comparatively higher MICs in our laboratory, the breakpoint interpretations of resistance and sensitivity of the same isolates at both laboratories were similar. GC agar is currently in use for both disc diffusion and Etest assays in a continuation of this surveillance study which was re initiated in 2018. So far, the resistance rates to tetracycline and ciprofloxacin are 100%, but a reduced resistance rate to penicillin has been observed. Chocolate agar is used in poorly resourced laboratories for susceptibility testing of *N*. *gonorrhoeae*.

## Conclusions

Rapidly emerging resistance to gonorrhea therapeutics traditionally used in Ghana raises a concern for resistance patterns and mutations which will need to be identified in real-time. Ongoing extensive nationwide surveillance is needed in Ghana to further elucidate AMR trends in an under-sampled area of the world. In response to these needs, deep sequencing is planned for the nucleic acid extracts of all isolates that were examined in this study as well as additional isolates that have been collected since June 2018. The results will hopefully fill the gap and address the shortcomings of the present study, as we also look forward to expanding the surveillance on a nationwide scale.

## Supporting information

S1 Table AMR of 44 isolates with inhibition zone size and MIC data. (PDF)

S2 Table Molecular worksheet for 44 isolates. (PDF)
