# Supplementary material for: Antimicrobial resistance (AMR) and molecular characterization of Neisseria gonorrhoeae in Ghana, 2012-2015
Source: PLoS One. 2019 Oct 10;14(10):e0223598. doi: 10.1371/journal.pone.0223598 (PMC6786528; doi:10.1371/journal.pone.0223598)
Supplement: S1 Table — (PDF) [file pone.0223598.s001.pdf]

ID	Site	Species ID	Pen	IHZ_Pen	MIC_Pen_Etest	Te	IHZ_Te	MIC_Te_Etest	AZ	IHZ_AZ	MIC_AZ_Etest	TXL	IHZ_TXL	MIC_TXL_Etest	IX	IHZ_IX	MIC_IX_Etest	CL	IHZ_CL	MIC_CL_Etest	SP	IHZ_SP	MIC_SP_Etest	Penicillinase by API-NH
STI-ADB-055 (2)	ACCRA	Neisseria gonor	Intermediate	46 [I]	0.94[I]	Resistant	22.3[R]	4[R]	Susceptible	39.9[S]		Susceptible	56.9[S]		Susceptible	45.9[S]		Resistant	13.3[R]	4[R]	Susceptible	27.9[S]		negative
STI-ADB-088(4)	ACCRA	Neisseria gonor	Resistant	6 [R]	>32[R]	Resistant	6[R]	128[R]	Susceptible	29[R]	0.75[S]	Susceptible	39.3[S]		Susceptible	34.9[S]		Resistant	6[R]	>32[R]	Susceptible	24.4[S]		positive
STI-ADB-095(3)	ACCRA	Neisseria gonor	Resistant	6 [R]	>32[R]	Resistant	13.9[R]	32[R]	Susceptible	25[R]	0.094[S]	Susceptible	25.7[R]	?	Susceptible	23.8[R]	0.094[S]	Susceptible	30.8[I]	0.006[S]	Susceptible	16.8[I]	12[S]	positive
STI-ADB-099(5)	ACCRA	Neisseria gonor	Resistant	6 [R]	>32[R]	Resistant	6[R]	128[R]	Susceptible	29.7[R]	?	Susceptible	42.7[S]		Susceptible	36.1[S]		Resistant	8.9[R]	12[R]	Susceptible	23.9[S]		positive
STI-ADB-129(6)	ACCRA	neisseria gonor	Resistant	10.6[R]	>32[R]	Resistant	8.6[R]	16[R]	Susceptible	42.3[S]		Susceptible	52.5[S]		Susceptible	49.7[S]		Resistant	13.1[R]	4[R]	Susceptible	36.1[18]		positive
STI-ADB-135(9)	ACCRA	Neisseria spp	Resistant	6 [R]	>32[R]	Resistant	9.3[R]	32[R]	Susceptible?	27.3[R]	?	Susceptible	38.3[S]		Susceptible	36.6[S]		Intermediate	11.1[R]	0.12[I]	Susceptible		8[S]	positive
STI-ADB-191(7)	ACCRA	neisseria gonor	Resistant	13[R]	>32[R]	Resistant	14[R]	24[R]	Susceptible	34[S]		Susceptible?	35[R]	?	Susceptible	36.3[S]		Resistant	17.9[R]	4[R]	Susceptible		8[S]	negative
STI-ADB-208(10)	ACCRA	neisseria gonor	Resistant	6 [R]	>32[R]	Resistant	6[R]	128[R]	Susceptible	30.3[S]		Susceptible	40[S]		Susceptible	36.3[S]		RESISTANT	6[R]	32[R]	Susceptible		12[S]	positive
STI-ADB-216(11)	ACCRA	neisseria gonor	Resistant	6 [R]	>32[R]	Resistant	12.3[R]	32[R]	Susceptible	36[S]		Susceptible	43[S]		Susceptible	34.4[S]		Susceptible	40.9[I]	0.008[S]	Susceptible		8[S]	positive
STI-ADB-226(12)	ACCRA	neisseria gonor	Resistant	12.3[R]	4[R]	Resistant	24[R]	10.9[R]	Susceptible	20.8[R]	0.094[S]	Susceptible	39.3[S]		Susceptible	32.3[S]		Resistant	8.2[R]	12[R]	Susceptible		8[S]	positive
STI-ADB-230(13)	ACCRA	neisseria gonor	Resistant	26.9[I]	4[R]	INTERMEDIATE	25.6[R]	2[R]	SUSCEPTIBLE	29.4[R]	0.38[S]	Susceptible	35.2[S]		Resistant	29.2[R]	0.75[R]	Resistant	6[R]	>32[R]	Susceptible		16[S]	negative
STI-ADB-242(14)	ACCRA	Neisseria spp	Resistant	6 [R]	>32[R]	Resistant	6[R]	48[R]	Susceptible	31.8[S]		Susceptible	38.7[S]		Susceptible	34.2[S]		Resistant	6[R]	>32[R]	Susceptible		8[S]	positive
STI-AIR-015(37)	TAKORADI	Neisseria spp	Resistant	15.8[R]	8[R]	Resistant	11.2[R]	24[R]	Susceptible	32.5[S]		Susceptible	45.8[S]		Susceptible	40.1[S]		Resistant	14.4[R]	6[R]	Susceptible		8[S]	positive
STI-ARM-046(15)	TAKORADI	Neisseria gonor	Resistant	6 [R]	>32[R]	Resistant	12[R]	32[R]	Susceptible	32.3[S]		Susceptible	40.3[S]		Susceptible	34.4[S]		Resistant	13.6[R]	16[R]	Susceptible	24.8[S]		negative
STI-ARM-069(16)	TAKORADI	Neisseria spp	Resistant	6 [R]	>32[R]	Resistant	20[R]	16[R]	Susceptible	34.5[S]		Susceptible?	33.6[R]	?	Susceptible	6[R]	0.016[S]	Susceptible	22.7[R]	0.006[S]	Susceptible	29.6[S]		positive
STI-ARM-073(17)	TAKORADI	neisseria gonor	Resistant	9.5[R]	>32[R]	Resistant	10.2[R]	32[R]	Susceptible	35.5[S]		Susceptible	48.4[S]		Susceptible	42.8[S]		Susceptible	48.4[S]		Susceptible	25.6[S]		positive
STI-ARM-103(18)	TAKORADI	Neisseria gonor	Intermediate	42.1[I]	0.19[I]	Resistant	11.8[R]	32[R]	Susceptible	36.5[S]		Susceptible	49.3[S]		Susceptible	40.3[S]		Resistant	17.4[R]	4[R]	Susceptible	28.4[S]		negative
STI-ARM-106(19)	TAKORADI	neisseria gonor	Resistant	6 [R]	>32[R]	Resistant	6[R]	128[R]	Susceptible	33.2[S]		Susceptible	46.8[S]		Susceptible	39.7[S]		Resistant	6[R]	>32[R]	Susceptible	26.2[S]		positive
STI-ARM-122(20)	TAKORADI	Neisseria spp	Resistant	6 [R]	>32[R]	Resistant	14.1[R]	16[R]	Susceptible	31.7[S]		Susceptible	48.3[S]		Susceptible	39.1[S]		Resistant	15.2[R]	3[R]	Susceptible		4[S]	positive
STI-ARM-127(21)	TAKORADI	Neisseria spp	Resistant	9.1[R]	>32[R]	Resistant	9.4[R]	48[R]	Susceptible	25.5[R]	?	Susceptible	41.4[S]		Susceptible	37.1[S]		Susceptible	36.7[I]	0.016[I]	Susceptible		12[S]	positive
STI-ARM-162(22)	TAKORADI	neisseria gonor	Resistant	6 [R]	>32[R]	Resistant	22.4[R]	3[R]	Susceptible	30.5[S]		Susceptible	37.1[S]		Susceptible	36[S]		Resistant	6[R]	>32[R]	Susceptible		12[S]	positive
STI-ARM-163(23)	TAKORADI	neisseria gonor	Resistant	6 [R]	>32[R]	Resistant	20.6[R]	3[R]	SUSCEPTIBLE	27.1[R]	0.38[S]	Susceptible	36.6[S]		Susceptible	29.9[R]	?	Resistant	6[R]	16[R]	Susceptible		16[S]	positive
STI-ARM-165(24)	TAKORADI	neisseria gonor	Resistant	6 [R]	>32[R]	Resistant	22[R]	3[R]	Susceptible	30.4[S]		Susceptible	37.7[S]		Susceptible	33.8[S]		Resistant	6[R]	12[R]	Susceptible		12[S]	positive
STI-ARM-212(25)	TAKORADI	Neisseria gonor	Intermidia	25[R]	0.75[I]	Resistant	16[R]	16[R]	Susceptible	30[R]	?	Susceptible?	32[R]	?	Susceptible	34[S]		Resistant	15[R]	6[R]	Susceptible	27[S]	4[S]	negative
STI-NAV-020(26)	TAKORADI	neisseria gonor	Resistant	7[R]	3[R]	Resistant	15.8[R]	12[R]	Susceptible	40.8[S]		Susceptible	39[S]		Susceptible	44.2S]		Resistant	15.4[R]	>32[R]	Susceptible	29[S]		positive
STI-NAV-029(27)	TAKORADI	neisseria gonor	Resistant	6 [R]	>32[R]	Resistant	6[R]	128[R]	Susceptible	30.8[S]	0.5[S]	Susceptible	47.5[S]		Susceptible	43.6[S]		Resistant	6[R]	>32[R]	Susceptible	27.9[S]		positive
STI-NAV-031(28)	TAKORADI	neisseria gonor	Resistant	6 [R]	>32[R]	Resistant	6[R]	256[R]	Susceptible?	27.8[R]	?	Susceptible	46.9[S]		Susceptible	42[S]		Resistant	6[R]	>32[R]	Susceptible	26.1[S]		positive
STI-NAV-040(29)	TAKORADI	neisseria gonor	Resistant	10.6[R]	>32[R]	Resistant	10.6[R]	24[R]	Susceptible	39[S]		Susceptible	42[S]		Susceptible	38.8[S]		Resistant	15.3[R]	3[R]	Susceptible	26.7[S]		positive
STI-TMH-040(30)	ACCRA	Neisseria gonor	Resistant	10.6[R]	24[R]	Resistant	10.3[R]	256[R]	Susceptible	39[S]		Susceptible	42[S]		Susceptible	38.8[S]		Susceptible	15.3[R]	0.004[S]	Susceptible	26.7[S]		negative
STI-TMH-042(31)	ACCRA	Neisseria gonor	Resistant	6 [R]	>32[R]	Resistant	14[R]	24[R]	Susceptible	35.1[S]	0.094[S]	Susceptible	35[R]	?	Susceptible	32[S]		Resistant	17[R]	8[R]	Susceptible	26[S]		positive
STI-TMH-050(32)	ACCRA	Neisseria gonor	Resistant	6 [R]	>32[R]	Resistant	15[R]	24[R]	Susceptible	30.7[S]	0.125[S]	Susceptible	41[S]		Susceptible	35[S]		Resistant	15[R]	8[R]	Susceptible	26[S]		positive
STI-TMH-067(33)	ACCRA	Neisseria gonor	Resistant	6 [R]	>32[R]	Resistant	13.7[R]	24[R]	Susceptible	35.4[S]		Susceptible	43.2[S]		Susceptible	40.8[S]		Resistant	16.1[R]	3[R]	Susceptible	23.7[S]		positive
STI-TMH-068(34)	ACCRA	Neisseria gonor	Resistant	6 [R]	>32[R]	Resistant	6[R]	128[R]	Susceptible	27.8[R]	0.125[S]	Susceptible	33.3[R]	0.023[S]	Susceptible	32.7[S]		Resistant	6[R]	>32[R]	Susceptible	21[S]		positive
STI-TMH-074(35)	ACCRA	Neisseria spp	Resistant	6.7[R]	>32[R]	Resistant	11.9[R]	32[R]	Susceptible	26.4[R]	0.125[S]	Susceptible	31.9[R]	0.004[S]	Susceptible	32[S]		Resistant	11.4[R]	6[R]	Susceptible	20.5[S]		positive
STI-TMH-289(36)	ACCRA	neisseria gonor	Resistant	6 [R]	>32[R]	Resistant	6[R]	128[R]	Susceptible	28[R]	0.19[S]	Susceptible	36.8[S]		Susceptible	34.4[S]		Resistant	6[R]	32[R]	Susceptible	21[S]		positive
STI-TMH-384(39)	ACCRA	Neisseria gonor	Intermediate	34.6[I]	0.75[I]	Resistant	6[R]	96[R]	Susceptible	34.6[S]		Susceptible	49.8[S]		Susceptible	42.7[S]		Resistant	6[R]	>32[R]	Susceptible		12[S]	negative
STI-TMH-453(40)	ACCRA	Neisseria gonor	Resistant	10.9[R]	>32[R]	Resistant	11.7[R]	24[R]	Susceptible	33[S]		Susceptible	39.9[S]		Susceptible	37.7[S]		Resistant	14.1[R]	4[R]	Susceptible		6[S]	positive
STI-TMH-455(41)	ACCRA	Neisseria gonor	Resistant	6 [R]	>32[R]	Resistant	12.7[R]	12[R]	Susceptible	38.2[S]		Susceptible	52.8[S]		Susceptible	41.1[S]		Resistant	14.5[R]	4[R]	Susceptible		3[S]	positive
STI-TMH-458(42)	ACCRA	Neisseria gonor	Resistant	6 [R]	>32[R]	Resistant	17.8[R]	16[R]	Susceptible	31.9[S]		Susceptible	44.1[S]		Susceptible	37.9[S]		Resistant	11.2[R]	16[R]	Susceptible		24[S]	positive
STI-TMH-462(43)	ACCRA	neisseria gonor	Resistant	6 [R]	>32[R]	Resistant	14.5[R]	24[R]	Susceptible	32.7[S]		Susceptible	39.7[S]		Susceptible	38.2[S]		Resistant	11.2[R]	16[R]	Susceptible		24[S]	positive
STI-TMH-465(44)	ACCRA	Neisseria gonor	Resistant	6 [R]	>32[R]	Resistant	6[R]	96[R]	Susceptible	33.2[S]		Susceptible	42.2[S]		Susceptible	38.7[S]		Susceptible	44.9[S]	0.016[S]	Susceptible		12[S]	positive
STI-TMH-484(45)	ACCRA	Neisseria spp	Resistant	6 [R]	>32[R]	Resistant	13.6[R]	16[R]	Susceptible	38.6[S]		Susceptible	45.7[S]		Susceptible	44.4[S]		Resistant	15.8[R]	6[R]	Susceptible		8[S]	positive
STI-TMH-524(46)	ACCRA	Neisseria gonor	Resistant	6 [R]	>32[R]	Resistant	11.3[R]	32[R]	Susceptible	30.2[S]	0.094[S]	Susceptible	30.9[R]	?	Susceptible	23.5[R]	0.125[S]	Resistant	10.2[R]	8[R]	Susceptible		16[S]	positive
STI-TMH-537(47)	ACCRA	Neisseria gonor	Resistant	6 [R]	>32[R]	Resistant	12.8[R]	24[R]	Susceptible	28.1[R]	0.19[S]	Susceptible	37.7[S]		Susceptible	37.7[S]		Resistant	14.2[R]	3[R]	Susceptible	22.1[S]		positive
