# Supplementary material for: Antimicrobial resistance (AMR) and molecular characterization of Neisseria gonorrhoeae in Ghana, 2012-2015
Source: PLoS One. 2019 Oct 10;14(10):e0223598. doi: 10.1371/journal.pone.0223598 (PMC6786528; doi:10.1371/journal.pone.0223598)
Supplement: S2 Table — (PDF) [file pone.0223598.s002.pdf]

	Molecular work performed on Neisseria gonorrhoeae isolates									
			Test performed							
Sequencing primers			Primer pair 1	Primer pair 2	Primer pair 3a,3b,3c,3d	Primer pair 3a, 3b,3c,3d	Por allele #	TbpB allele #	Allelic profile	
Serial number	Sample number	Collection date	Por PCR 680bp	TbpB PCR 550bp	PBP2 PenA1 PCR 1100bp	PBP2 PenA2 PCR 1200bp			(Sequence type)	beta lactamase reactivity
2	ADB055	20-Jan-14	1	1	0	1	1050	98	1737	neg
3	ADB095	14-Apr-14	1	1	0	1	90	3608	16216	pos
4	ADB088	27-Mar-14	1	1	0	1	9431	2609	16217	pos
5	ADB099	5-May-14	1	1	0	1	315	996	8948	pos
6	ADB129	31-Jul-14	1	1	0	1	315	1649	16218	pos
7	ADB191	21-Apr-15	1	1	0	1	90	133	355	neg
9	ADB135	12-Aug-14	1	1	0	1	596	2610	16219	pos
10	ADB208	13-May-15	1	1	0	1	315	996	8948	pos
11	ADB216	10-Jun-15	1	1	0	1	1923	29	3178	pos
12	ADB226	29-Jun-15	1	1	0	1	64	1844	10251	pos
13	ADB230	12-Aug-15	1	1	1	1	908	110	1407	neg
14	ADB242	14-Sep-15	1	1	0	1	9432	58	16220	pos
15	ARM046	20-Feb-13	1	1	0	1	9433	1134	16221	neg
16	ARM069	13-Aug-13	1	1	0	1	9434	1649	16222	pos
17	ARM073	10-Sep-13	1	1	0	1	9434	1649	16222	pos
18	ARM103	21-Feb-14	1	1	0	1	5861	98	9919	neg
19	ARM106		1	1	0	1	315	996	8948	pos
20	ARM122	5-Jun-14	1	1	0	1	1050	98	1737	pos
21	ARM127	26-Jun-14	1	1	0	1	90	732	3370	pos
22	ARM162	26-Jun-15	1	1	0	1	315	996	8948	pos
23	ARM163	23-Jun-15	1	1	0	1	315	996	8948	pos
24	ARM165	3-Jul-15	1	1	0	1	315	996	8948	pos
25	ARM212	1-Dec-15	1	1	0	1	9435	58	16223	neg
26	NAV020	9-Aug-13	1	1	0	1	9436	98	16224	pos
27	NAV029	25-Feb-14	1	1	0	1	315	996	8948	pos
28	NAV031	26-Feb-14	1	1	0	1	315	996	8948	pos
29	NAV040	26-Nov-13	1	1	0	1	90	133	355	pos
30	TMH040	12-Sep-12	1	1	0	1	4301	131	16225	neg
31	TMH042	13-Sep-12	1	1	0	1	9437	98	16226	pos
32	TMH050	27-Sep-12	1	1	0	1	596	98	16227	pos
33	TMH067	29-Oct-12	1	1	0	1	9437	98	16226	pos
34	TMH068	6-Nov-12	1	1	0	1	315	996	8948	pos
35	TMH074	9-Nov-12	1	1	0	1	9438	98	16228	pos
36	TMH289	11-Feb-14	1	1	0	1	315	2611	16229	pos
37	AIR015		1	1	0	1	90	732	3370	pos
39	TMH384	21-Aug-14	1	1	0	1	1350	996	16230	neg
40	TMH453	26-Jun-15	1	1	0	1	90	133	355	pos
41	TMH455	29-Jun-15	1	1	0	1	832	2612	16231	pos
42	TMH458	3-Jul-15	1	1	0	1	64	1844	10251	pos
43	TMH462	7-Jul-15	1	1	0	1	64	1844	10251	pos
44	TMH465	16-Jul-15	1	1	0	1	3611	119	9523	pos
45	TMH484	20-Jun-15	1	1	1	1	1439	2613	16232	pos
46	TMH524	30-Oct-15	1	1	0	1	1923	191	12791	pos
47	TMH537	1-Dec-15	1	1	0	1	103	98	2025	pos
